# Reversing Type 2 Diabetes in a Primary Care-Anchored eHealth Lifestyle Coaching Programme in Denmark: A Randomised Controlled Trial

**DOI:** 10.3390/nu14163424

**Published:** 2022-08-19

**Authors:** Jeanette Reffstrup Christensen, Ditte Hjorth Laursen, Jørgen Trankjær Lauridsen, Laura Hesseldal, Pernille Ravn Jakobsen, Jesper Bo Nielsen, Jens Søndergaard, Carl J. Brandt

**Affiliations:** 1 Research Unit of General Practice, Department of Public Health, The Faculty of Health Sciences, University of Southern Denmark, 5000 Odense, Denmark; 2 User Perspectives and Community-Based Interventions, Department of Public Health, The Faculty of Health Sciences, University of Southern Denmark, 5000 Odense, Denmark; 3 The MOVE Unit, Research Unit of General Practice, The Faculty of Health, Aarhus University, 8000 Aarhus, Denmark; 4 Department of Public Health, Faculty of Health and Medical Science, University of Copenhagen, 2200 Copenhagen, Denmark; 5 Department of Economics, The Faculty of Business and Social Sciences, University of Southern Denmark, 5000 Odense, Denmark; 6 Liva Healthcare, 1434 Copenhagen, Denmark

**Keywords:** digital behavioural coaching, health behaviour change, interactive advice, lifestyle change, mobile intervention, obesity, quality of life, participant engagement, telemedicine

## Abstract

The goal of this trial was to investigate whether an eHealth lifestyle coaching programme led to significant weight loss and decreased Haemoglobin A1c (HbA1c) in patients with type 2 diabetes. In an RCT, 170 patients were enrolled from 2018 to 2019 for intervention or control. Inclusion criteria were diagnosed with type 2 diabetes, BMI 30–45 kg/m^2^, and aged 18–70 years. Exclusion criteria were lacks internet access, pregnant or planning a pregnancy, or has a serious disease. Primary and secondary outcomes were a reduction in body weight and HbA1c. At six months, 75 (75%) patients in the intervention group and 53 (76%) patients in the control group remained in the trial. The mean body weight loss was 4.2 kg (95% CI, −5.49; −2.98) in the intervention group and 1.5 kg (95% CI, −2.57; −0.48) in the control group (*p* = 0.005). In the intervention group, 24 out of 62 patients with elevated HbA1c at baseline (39%) had a normalized HbA1c < 6.5% at six months, compared to 8 out of 40 patients with elevated HbA1c at baseline (20%) in the control group (*p* = 0.047). The eHealth lifestyle coaching programme can lead to significant weight loss and decreased HbA1c among patients with type 2 diabetes, compared to standard care.

## 1. Introduction

With the incidence of overweight and obesity rapidly increasing in adults, the prevalence of chronic lifestyle-related diseases such as type 2 diabetes (T2D) is also increasing, and cost-effective management programmes for both overweight and T2D are needed [1]. In 2017, approximately 462 million individuals were affected by T2D, corresponding to 6% of the world’s population or a prevalence of 6000 cases per 100,000 [2]. Over 1 million deaths per year can be attributed to diabetes alone, making it the ninth leading cause of mortality. The global prevalence of T2D is projected to increase to approximately 7% by 2030, reflecting a continued rise across all regions of the world [2]. Several studies have shown that people with T2D can return to normal glucose control by losing weight [3,4]. Haemoglobin A1c (HbA1c) is used to estimate to what degree T2D is under control. HbA1c shows the average blood glucose for patients over a period of two to three months [5]; therefore HbA1c holds the primary focus when evaluating cost-effective management programmes. Even a marginal weight reduction can slow down T2D progression, leading to remission for 46% to 54% of patients, defined as HbA1c < 6.5% [3,4]. The potential of interventions supporting healthier lifestyle behaviours to reduce the incidence of T2D is well-documented [6]. Although solid long-term evidence is still lacking, it seems that long-lasting lifestyle interventions can reduce complications and mortality over 30 years for patients with glucose intolerance [7].

Internet and mobile interventions aimed at healthy lifestyles have been given much attention due to their potential for privacy, user control, interactive advice, opportunities for real-time modifications, scalability, accessibility, and low cost [8,9]. Metanalyses and systematic reviews show that electronic health (eHealth) and mobile health (mHealth) solutions are effective at supporting healthier diets and weight loss, and increasing levels of physical activity [8,10,11,12]. The literature shows that the effectiveness of these solutions varies, and a critical component is the combination of behavioural change techniques (BCTs) and feedback [13]. Digital feedback can be automated, semi-automated, asynchronous, and/or face-to-face, delivered by a health coach. Solutions incorporating feedback and coaching provided by a health coach are most effective, and higher feedback frequency is associated with better outcomes [14,15].

### 1.1. Development of a New eHealth App and Its Effectiveness

Based on promising findings from the literature, a Danish eHealth app called LIVA 1.0 (long-term Lifestyle change InterVention and eHealth Application) was developed [16]. In an observational study, LIVA 1.0 demonstrated promising weight loss results in patients with T2D in real-life settings [16,17,18]. LIVA 1.0 was a browser-based solution. With LIVA 1.0, we found that, in a Danish setting, personal eHealth lifestyle coaching combined with various BCTs, such as self-monitoring, reminders, tailored information, personal feedback, and face-to-face support, led to a relevant weight loss of 5.1 kg during a 20-month period [19]. These results have been confirmed in a British municipality setting [9]. In the British randomized controlled trial (RCT), men with T2D lost an average of 5.4 kg compared to a 2.8 kg weight loss in a control group receiving standard care [9].

The current version of LIVA 2.0 was developed based on experiences from the Danish and British studies [9,19], as well as experiences of approximately 140,000 individuals who had used LIVA 1.0 in Denmark and Britain over a 15-year period. Its development was also based on three qualitative interview studies with a. patients who had been using LIVA 1.0 [20], b. general practitioners (GPs) [21], and c. eHealth coaches [22] who had used the first prototype of LIVA 2.0. A finding from these studies was that to enable optimised digital tailored individual coaching, a strong empathic relationship seems to be essential. [20,23]. This was built at a physical face-to-face meeting with an individual health coach and followed up each week in synchrony visual online meetings. This meant that the health coach and the patient still met face-to-face, but instead of physical meetings, they met online. During the first meeting, the patient was told that chemistry was very important. If the patient did not feel that the health coach could understand his or her challenges or they did not feel they were in good hands, they had the possibility to be appointed to another health coach. The patient could state this by calling or emailing the LIVA administration, which happened only a few times. By establishing a personal relationship outside the digital environment as part of LIVA 2.0, and by maintaining it through the platform with backend follow-up, the solution facilitates tailored care and sustained participant engagement over time, with limited continued health coach input throughout the process of successfully achieving and sustaining lifestyle change [20]. To the best of our knowledge, LIVA 2.0 is the only eHealth lifestyle app that provides this form of hybrid solution in a clinical setting for T2D.

### 1.2. Study Objectives

The RCT investigated whether individualised digital lifestyle coaching enabled by an eHealth and mHealth solution could increase health for T2D patients by supporting them to lose weight, decrease BMI and hip and waist circumference, and improve blood glucose management compared to a control group receiving standard care with 6-month follow-up.

## 2. Materials and Methods

### 2.1. Study Design

A single-blinded RCT was carried out from March 2019 to October 2020 in two of the five regions of Denmark: The Region of Southern Denmark, which includes 22 municipalities, and the Capital Region of Denmark, which includes 28 municipalities. The study was approved by the scientific committee of the Region of Southern Denmark (S-20170183G) and registered on clinicaltrials.gov (NCT03788915). All methods are described in detail in the study protocol [16] and are also briefly described here.

### 2.2. Participants, Eligibility Criteria, and Recruitment Procedure

Each municipality in question recruited patients through advertising campaigns in local newspapers, on Facebook, and other social media platforms which were used in the local community. Patients were also recruited through general practices and patient organisations. Patients who were interested in participating could register their contact details on the LIVA 2.0 website [16]. These registered patients were subsequently contacted by telephone by a research assistant. The research assistant was responsible for screening each patient to assure that the patient in question was aligned with inclusion as well as exclusion criteria (as specified in Table 1).

Patients who were included in the trial received an email with detailed information about the study and an invitation to attend a baseline meeting with a research assistant. Patients who were excluded received an email with information about the standard care provided by their local municipality.

### 2.3. Baseline Meeting and Assessment

Patients who agreed to participate showed up for their baseline meeting where they provided their written informed consent and informed the research assistant about their current medication use. A brief medical examination was then performed (Table S1) which included measuring the patient’s height, weight, waist and hip circumference, and blood pressure [24]. Blood samples for HbA1c, total cholesterol, low-density lipoprotein cholesterol (LDL), high-density lipoprotein cholesterol (HDL), and triglycerides (TG) were also measured [25].

The patients then filled out an online questionnaire assessing sociodemographic data, physical exercise habits (how often the patients engaged in physical exercise that affected their breathing and routine exercise such as gardening), and diet habits (how often the patients consumed vegetables, fruit, fish, and sweets) (Table S2). The questionnaire also included the European Quality of Life—5 Dimensions scheme (EQ-5D-5L), and the Short-Warwick-Edinburgh Mental Well-being Scale (SWEMWBS) (Table S1) [26,27]. The EQ-5D-5L is a widely used generic preference-based health-related quality of life questionnaire. EQ-5D-5L has been widely used in diabetic populations as this population often reports lower scores compared to the general population [26]. There has been an increase in interest in the concept of mental well-being in the last ten years due to the recognition of its impact on individual general well-being. The WEMWBS was developed to meet the need for a psychometrically robust measurement that would enable the monitoring of mental well-being in the general population but also in the evaluation of projects and policies that aim to improve mental well-being [27]. The scale has been translated into more than 25 languages, including Danish, which was the version used. The short version of SWEMWBS was applied to reduce the burden on participants by not having to answer too many questions [27].

### 2.4. Randomisation and Blinding

After patients had completed the medical examination and filled out the questionnaires, they were randomised via an automated computer algorithm, making sure that the baseline examinations were completed while patients and research assistants were still blinded. Randomisation occurred in groups of 10 at a ratio of 6 (intervention group—digital lifestyle):4 (control group—standard care). Blinding the patients, the research assistant, and the health coach who provided lifestyle coaching were not possible after randomisation. The research assistant and health coach had no role in analysing or interpreting data. The patients’ GPs, who managed medications to control blood glucose, lipids, and blood pressure were not informed of the allocation group. The GPs handled medication in accordance with standard guidelines and practices for medication management based on quarterly standard care visits [28]. Some data used in the present study were retrieved from the electronic patient record without informing the GPs about the patient’s group allocation.

Regardless of whether patients were randomised to intervention or control groups, they maintained their usual contact with their GPs. This also meant that their GPs followed all recommended guidelines for their patients. For diabetes patients, the standard care setup in Denmark includes quarterly visits to the GP measuring HbA1c, blood pressure, lipids, and weight, apart from other relevant parameters in relation to the individual patient’s symptoms. Annually, the GP visits also include measuring albumin creatinine ratio, vitamin B12, and thyroid stimulating hormone [28].

### 2.5. Intervention Group

All patients randomised in the intervention group met with a health coach after the medical examination. The intervention group received the individualised digital lifestyle coaching LIVA 2.0 programme, described in the Template of the Intervention Description and Replication (TIDieR checklist) (Table S3) [16,17,18]. Briefly, the programme began with a one-hour face-to-face motivational interviewing session with a health coach. After the first personal meeting, the same health coach was coaching the patient throughout the period. If the health coach went on vacation or was sick for a short period, coaching was postponed. If the health coach was sick long-term, a personal meeting with a new health coach was arranged to secure a personal relationship, after which, the new health coach stayed with the patient for the rest of the period. Subsequently, at the first meeting, patients received login credentials for the LIVA 2.0 app and the health coach introduced the programme. Each patient and their health coach discussed and agreed on goals for diet, physical exercise, sleep, and any other relevant lifestyle areas that the patient was motivated to improve. The health coach was required to identify what health initiatives would benefit the patient the most based on the participant’s own wishes and to find out what was possible and realistic for the patient. The health coach considered both the patient’s personal barriers and facilitators. Using LIVA 2.0, patients completed daily records for the different selected goals and sent comments, concerns, and questions directly to their individual health coach who had access to all their patients’ profiles. The health coach provided weekly individualised synchronous or asynchronous online coaching, based on each of their patients’ input, encouraging and commending goal attainment and seeking to help patients remain motivated. The weekly coaching went on for the first three months, then biweekly for the next three months. The online meetings were cancelled only if the patients felt very sick, otherwise, they were merely postponed for a few days if needed. It was an advantage that the patients did not have to show up physically for the sessions but could meet with their health coach even if they felt a little unwell. Thus, only a few meetings were not carried out as planned.

The intervention included a high degree of behaviour change techniques (BCT) based on the Coventry, Aberdeen, and LOndon-REfined (CALO-RE) taxonomy. This meant providing information on the consequences of the behaviour in general and to the individual goal setting, behaviour and outcome, action planning, and barrier identification and problem solving; setting of graded tasks; prompt review of behavioural goals; prompt review of outcome goals; prompt rewards contingent on effort or progress toward behaviour; prompt generalisation of a target behaviour; and providing feedback on performance.

Goal setting was based on the SMART—Specific, Measurable, Attainable, Relevant, Timely model [29]. An example of a specific goal is ‘Choose one third of a plate of non-starchy vegetables with lunch and dinner’. This could be measured by sharing a picture with the health coach of meals over a specific period. To understand if a goal was attainable, the patient and health coach considered the patient’s other daily tasks and then determined if it was realistic in combination with all other tasks. When determining relevance, patients rated the goal on a scale from 0–10, with 10 being the most relevant. Timeliness was assessed with questions such as, ‘When can you take the first steps towards this goal?’ and ‘When would you like to have achieved this goal?’ Using the SMART goals in coaching ensured that patients could help define the goals themselves and had a clear plan for achieving their goals over a certain period.

### 2.6. Control Group

All patients randomised in the control group were invited to follow-up examinations at the same frequency as the intervention group. At the first examination, and after they were randomised in the control group, they were advised to contact their GP who could provide guidance about their health problems and further refer them to diabetes programmes in their municipalities. The municipality programmes included education about diet, exercise, and different forms of BCT, but because those components are not standardised, it results in a high degree of heterogeneity in terms of programme content, delivery structure, contacts to health professionals, and dose in relation to Danish municipality health promotion programmes [30]. The control group did not have access to the app, nor did they receive any digital interventions from LIVA 2.0.

### 2.7. Health Coaches

The health coaches providing the LIVA 2.0 programme were all educated as nurses, physiotherapists, dieticians, or occupational therapists. In Denmark, education for all four professions consists of 420 European Credit Transfer System (ECTS) points (3.5 years of full-time education). In addition to their education as healthcare professionals, all the health coaches received special training in how to use digital health coaching and had practiced municipal digital health coaching for at least two years. Each patient had a primary health coach, creating the opportunity to form a close and trusting relationship. Each patient also had a ‘back-up’ health coach if the primary health coach had a sick day or was on vacation.

### 2.8. Six-Month Assessment

After six months, patients were assessed by a research assistant again, undergoing a brief medical examination like the baseline examination. Patients informed the research assistant about their current medication use and if it had changed over the last six months. Patients were also asked to complete the same web-based questionnaires comprising sociodemographic data, exercise, and diet habits, the EQ-5D-5L, and the SWEMWBS assessments (Table S1) [26,27].

### 2.9. Outcomes

The primary outcome was mean body weight. The proportion of patients who lost >3%, >5%, and >10% of baseline body weight was also assessed. In accordance with Jensen et al. diabetes patients seem to start to benefit from weight loss at 3% [31]. The graded evidence statements that resulted from this effort provide the strongest support for weight loss beginning at 3% (for glycaemic measures and triglycerides) and 5% (for blood pressure and HDL and LDL cholesterol) to be considered clinically meaningful [31,32]. Secondary outcomes were mean HbA1c from baseline to six months and the proportion of patients whose HbA1c decreased or normalised to <6.5% at six months. Other outcomes included changes in body composition (BMI and hip waist ratio), lipids (total cholesterol, LDL, HDL, and TG), and systolic and diastolic blood pressure, as well as changes from baseline to six months in social demography, exercise and diet habits, and quality of life and mental well-being. In addition, changes over time in the use of medications to lower blood glucose, blood pressure, and cholesterol were assessed (Table 2). Information about medication use was retrieved at the baseline medical examination and the six-month medical examination.

Medication changes were evaluated by two authors with medical degrees (C.J.B. and J.S.) to assess if patients had reduced, stopped, started, or increased their use of medications.

### 2.10. Lost to Follow-Up

Patients were contacted up to four times by telephone in the week before their six-month appointments. If they did not respond, a voicemail was left explaining the purpose of the call. Another telephone call was made a week later and again one month later. Patients who had not responded after a month were considered lost to follow-up. In addition, patients whose assessments occurred later than 7.5 months after the baseline examination were not included in the six-month analysis.

### 2.11. Power

Based on a recent study evaluating a web-based weight loss intervention programme among men with diabetes, a mean weight loss of ≥4.5 kg over six months was expected in the intervention group, compared with 2.5 kg in the control group [9]. Detecting a 2 kg between-group difference in the primary outcome of body weight with 95% power and a 5% significance level required 55 patients in the intervention group and 32 patients in the control group, assuming a 6:4 allocation ratio [16]. The allocation assumption is to allow for dropout according to experienced attrition rates (39% in the intervention group and 57% in the control group are expected to drop out at 12 months), which is why we recruited 100 participants in the intervention group and 70 in the control group [9]. The standard deviation was assumed to be 5.6% [9]. Oversampling allowed for dropout rates as high as 45% in the intervention group and 55% in the control group, based on attrition rates in the reference study [9].

### 2.12. Statistical Analysis

Baseline characteristics of all patients allocated to the intervention and control groups were analysed using descriptive statistics. The statistical significance of differences in baseline characteristics of patients remaining in these groups at six months was assessed with the Student’s t-test for equal means or Chi-square table tests. To assess the statistical significance of between-group differences in body weight, HbA1c, medication use, exercise and diet habits, quality of life, and mental wellbeing at six months, a per protocol (PP) and an intention-to-treat analysis (ITT) were conducted. A PP analysis was performed along with the ITT to show how patients, per protocol, used the complex intervention specifically [33]. The statistical significance of between-group differences in all outcomes at six months was assessed with Student’s t-tests.

## 3. Results

### 3.1. Patient Characteristics

From March 2018 to March 2019, 170 patients were randomised: 100 (49 females) in the intervention group receiving digital lifestyle coaching and 70 (32 females) in the control group receiving standard care. The mean age of patients was 56 years, mean HbA1c was 7.4%, and mean body weight was 104 kg (Table 3).

**Figure 1:**
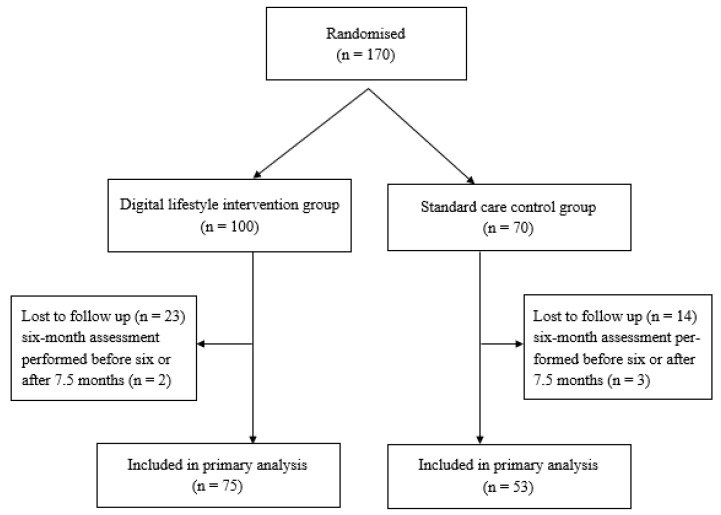
Participant flow diagram.

Patients were not included in the analysis at six months if they were lost to follow-up or had an assessment earlier than six months or later than 7.5 months after baseline examination. The interval was selected to give a relevant impression of the first six months of the intervention to patients typically seen by their GP every third month. At six months (range, 6–7.5 months), 128 patients were assessed: 75 (35 female) in the intervention group and 53 (23 female) in the control group (Figure 1).

Baseline characteristics including age, gender, glycaemic control, lipids, blood pressure, body composition, medication, quality of life, mental well-being, physical exercise, and dietary intake of patients remaining in the intervention and control groups did not differ at six months (Table 3). No differences were found between the intervention and the control group or between patients remaining in the intervention or who were dropping out except from systolic and diastolic blood pressure. Both diastolic and systolic were higher at baseline in patients dropping out (141.8 mmHg and 90.5 mmHg) than in patients remaining in the study (134.6 mmHg and 86.5 mmHg) (*p* = 0.01).

### 3.2. Weight

For per protocol patients, after completing six months of participation, the mean reduction in body weight was 4.24 kg in the intervention group vs. 1.52 kg in the control group (*p* = 0.005) (Table 4). In the intervention group, 36 (52%) patients lost >3% body weight, compared to 8 (22%) patients in the control group (*p* = 0.002). Similarly, 23 (33%) patients in the intervention group lost >5% body weight, compared to 4 patients (11%) in the control group (*p* = 0.011), and 8 patients (12%) lost >10% body weight in the intervention group vs. none in the control group (*p* = 0.031).

For patients in the ITT design, the mean reduction in body weight was 2.92 kg (3.87 to 1.98) for the intervention group vs. 0.81 kg (1.38 to 0.23) for the control group (*p* = 0.001). A 3%, 5%, and 10% weight loss were also significant (*p =* 0.000, 0.002, and 0.015, respectively).

### 3.3. HbA1c

For per protocol patients, HbA1c levels were reduced by 0.76% (−1.02; −0.49) in the intervention group and 0.61% (−0.85; −0.37) in the control group, which was not statistically significant. In the intervention group, 24 out of 62 (39%) patients with elevated HbA1c at baseline had normalised their HbA1c < 6.5% at six months, compared to 8 out of 40 (20%) patients in the control group with elevated HbA1c at baseline (*p* = 0.047). Patients in the intervention group whose HbA1c was increased at baseline but normalised at six months lost 7.38 kg on average, compared to 1.54 kg for the control group, a statistically significant difference (95% CI, −10.89; –0.79; *p* = 0.033). Patients in the intervention group with HbA1c < 6.5% both at baseline and at six months lost 6.04 kg on average, compared to 1.01 kg among the control group. The 5.03 kg difference was statistically significant (95% CI, −9.07; −0.98; *p* = 0.019).

### 3.4. Body Composition and Lipids

Mean BMI decreased significantly in the intervention group by 1.40 kg/m^2^ (95% CI, −1.81; −0.98), compared to 0.51 kg/m^2^ (95% CI, −0.85; −0.17, *p* = 0.005) in the control group. Hip and waist circumference also decreased significantly in the intervention group, compared to the control group (Table 4). In the intervention group, the mean total cholesterol decreased by 0.32 mmol/mL (95% CI, −0.56; −0.08). In contrast, it increased by 0.06 mmol/mL in the control group (95% CI, −0.24; 0.37, *p* = 0.048). Mean HDL decreased by an additional 0.12 mmol/mL in the intervention group vs. the control group (95% CI, −0.22; −0.02, *p* = 0.024). No significant changes were observed in LDL, TG, or blood pressure.

### 3.5. Medication Use

Change in medication was measured by asking the patients at the six-month visit (*n* = 106) and by looking at the shared pharmacological registration data (Fælles medicinkort) (*n* = 22). Out of 74 patients who took glucose-lowering medication at baseline examination in the intervention group, 8 reduced and 3 stopped their medication while 2 increased their medication. Out of the 41 patients who took glucose-lowering medication in the control group, 1 patient reduced their medication, and none stopped their medication, while 7 increased their medication. Overall, 11 out of 74 (15%) patients in the intervention group compared to 1 (2%) in the control group reduced their glucose-lowering medication (*p =* 0.015). In total, 2 of 74 (3%) in the intervention group compared to 7 of 41 (17%) in the control group increased their use of glucose-lowering medication (*p =* 0.021). No significant changes were seen in the amount of lipid and blood pressure lowering medication.

### 3.6. Exercise Habits

Both patients in the intervention group and the control group registered increased average moderate physical exercise that affected their breath without any difference in the two groups (*p =* 0.600) Patients in the intervention group also increased their everyday physical activity, which was not the case for patients in the control group. The difference between groups was not significant (*p* = 0.210)

### 3.7. Dietary Habits

The intervention group increased both their vegetable and fruit consumption, which was not the case for the control group. The between group difference over time was only significant for fruit consumption (*p* = 0.04). Both groups increased their consumption of sweets, but without difference at six months.

### 3.8. Quality of Life and Mental Well-Being

Differences in changes from baseline to six months EQ-5D-5L and SWEMWBS scores were not statistically significant (Table 4).

## 4. Discussion

### 4.1. Principal Findings

The primary objective of this study was to analyse if individualised digital lifestyle coaching enabled by an eHealth and mHealth solution could assist patients with T2D to lose weight and improve blood glucose control. We found that the LIVA app was an effective tool to both make T2D patients lose weight and improve blood glucose control when analysing data with PP and ITT statistical methods.

In the six-month RCT, the mean reduction in weight for patients in the intervention group was significantly higher compared to patients in the control group both in the PP and in the ITT analysis. The mean reduction in body weight for PP patients was 4.24 kg in the intervention group and 1.52 kg in the control group. The difference between the two groups over time was also significant in the ITT analysis, with a reduction of 2.92 kg for the intervention group and a reduction of 0.81 kg for the control group. These findings are in line with the findings in a systematic review of systematic reviews on eHealth interventions covering studies back to 2005 [34]. The reduction in body weight was 1.00 kg to 2.40 kg [35]. A more recent metanalysis, solely looking at studies with interventions using mobile devices, has shown a weight loss of 0.84 kg [35]. The present, more improved outcome compared to other studies is probably due to patients in the intervention group having received a combination of asynchronous eHealth coaching and face-to-face health coaching with different BCTs used, as also seen in other studies [15,18,20,21,22,35,36,37,38]. Our present intervention resulted in > 5% weight reduction for 33% of the intervention group, compared to 11% of patients in the control group, showing that a clinical impact on complications for patients with T2D could be expected in relation to later complications [33]. Despite the changes from 0–6 months in elevated HbA1c for both the intervention group (−0.76%) and control group (−0.61%), the group difference was not significant. The non-significant finding was unexpected, as significant differences were seen in a metanalysis by Wu et al. in 2018 [39]. Our non-significant findings could to some extent be explained by the increased use of medication in the control group as well as in the intervention group. A study from 2021 documents that T2D patients in standard care in Denmark are closely followed and well-treated [40]. This can also be an explanation for why the control group decreased HbA1c levels like the intervention group. We can only suspect that the LIVA intervention could have a higher impact in countries where T2D patients are not as closely followed. In the present study, we saw a significant reduction in glucose-lowering medication in the intervention group, while patients in the control group increased medication significantly. However, in accordance with Michaud et al., who found an HbA1c fall of 0.02% for every lost pound, the fall in HbA1c was slightly larger than what could be expected from weight loss alone [41]. This might explain why 39% of patients in the intervention group with elevated HbA1c at baseline had a normal HbA1c level at six months, compared to 20% of controls, giving an estimated effect on glycaemic control of a 0.30–0.50% higher reduction in HbA1c [41]. Body composition was reduced significantly between the groups over time, both regarding BMI, hip and waist circumference and hip/waist ratio. This reduction in hip/waist ratio is associated with a reduced risk of cardiovascular complications in T2D [42].

Total cholesterol was significantly improved in the intervention group (−0.32 mmol/mL) compared to an increase of 0.06 mmol/mL in the control group (*p* = 0.024). This significant difference was not seen in LDL, probably since LDL could not be analysed when the TG level was above 2.50. Additionally, while HDL was reduced significantly, the reduction in TG did not reach any significant level. No significant change was found in the change in cholesterol-lowering medication. This is interesting as it reduces the risk of complications and cardiovascular disease. These findings are in line with other app solutions leading to behavioural change, due, to some extent, to increased activity levels [43]. Even though the amount of moderate exercise did not differ in the two groups, routine exercise increased non-significantly in the intervention group. This is in line with Hamaja et al. from 2021 [43]. They failed in increasing moderate exercise but found an increase in step count [43].

Diet only changed marginally; although the intervention group may have started eating more vegetables and fruit, they did not change their consumption of fish and sweets. Even though the patients in the intervention group only changed their everyday physical activity and not their actual level of exercise, and increased their intake of fruit and vegetables, but did not change the rest of their dietary intake, it still affected their weight, body composition, and HbA1c significantly. Our assumption is that the patients have changed several minor lifestyle factors but that our subjective questionnaire had not been able to measure the minor lifestyle changes.

Blood pressure was reduced in the intervention group and in the control group, but no difference was found even though it seemed that the medication given did not change in either group. This is different from other interventions, which showed smaller reductions in blood pressure of 1.92 mmHg in the intervention group compared to a reduction of 0.23 mmHg in the control group [39].

Quality of life and mental well-being did not change in our study even though another study suggests that this can often be seen in eHealth interventions that provide coaching [42]. In a 12-week, four-armed RCT study by Bollyky et al. in 2018, patients received blood glucose monitoring with the addition of light or more intensive lifestyle coaching [44]. The study showed that the more intensive the coaching was, the more the participants lost weight and there was an additional decrease in HbA1c [44]. eHealth and mHealth lifestyle interventions shall continue to benefit from a lower intervention cost, but to be most effective, these interventions must still be individual, tailored, and of a certain intensity, and must contain an empathic relationship with a trusted health coach [28]. This also applies in the longer term if the effects are to be maintained. Without it being examined in the present study and solely based on the authors’ years of experience in developing and delivering lifestyle interventions, a few recommendations will be made. First, that the patients continue to register their lifestyle in the LIVA app for several years after their health goals have been reached. Second, that via the app, they can submit asynchronous questions, seek help, and expect a quick response. Third, that they can request synchronous coaching sessions with their individual health coach, with whom they have built a close relationship over time. Fourth, and finally, allow their individual health coach to reach out to the patients if they can see via the electronic records that one of their patients is having difficulty maintaining the new lifestyle. Whether the offer is to last for one or more years must be clarified in our future studies.

### 4.2. Limitations

Study limitations include the loss to follow-up or exclusion due to the timing of follow-up assessment of approximately 25% of patients at six months. We were also unable to do dropout analyses, as data protection rules prevented us from contacting participants who wanted to leave the study. We can therefore not comment on whether there are special personal characteristics that apply to whether one responds positively to an eHealth and mHealth intervention. It is thus possible that digital lifestyle interventions may only affect some people and not others. Thus, we want to explore this in a future study. However, the baseline characteristics of the remaining patients in the intervention and control groups did not differ significantly, indicating the results were not affected by selection bias. The six-month follow-up period, although consistent with other reports, does not support any conclusions about the longer-term effects of the intervention. The study design did not allow us to examine any possible dose-response relationships between intervention components (e.g., monitoring and coaching) or the intervention as a whole and the outcomes we observed. Finally, even though some of the most significant markers for the development of T2D are HbA1c level, overweight, dietary composition, and physical activity, which are all included in the present study, we could also have included, e.g., fasting plasma glucose levels or an oral glucose tolerance test. These are not considered in the trial, which would have been desirable.

## 5. Conclusions

The decreases in body weight, remission rate for hyperglycaemia, BMI, and hip and waist circumference can be improved after six months of use of the digital lifestyle coaching app, LIVA 2.0, for patients with T2D, thereby increasing their health. Further research is required to explore the longer-term effects of similar interventions.

## Figures and Tables

**Table 1:** Inclusion and exclusion criteria.

Inclusion criteria (1) Diagnosed with Type 2 Diabetes (2) Body mass index 30–45 kg/m^2^ (3) Aged 18–70 years Exclusion criteria (1) Lacks internet access via a computer or smartphone (2) Is pregnant or is planning a pregnancy soon (3) Has a serious or life-threatening disease with a life expectancy of less than one year

**Table 2:** Classification of changes in medication.

Four types of change in medicine were defined: (1) Starting new medication for a specific indication; (2) Increasing dosage or adding one or more new medications for an indication; (3) Reducing dosage or reducing one or more new medications for an indication; (4) Discontinuing a medication for the indication.

Glucose-lowering medications included biguanide, glucagon-like peptide-1 analogues, and insulin. Blood pressure lowering medications included diuretics, beta-blockers, ACE inhibitors, angiotensin II receptor blockers, calcium channel blockers, alpha-blockers, alpha-2 receptor agonists, central agonists, peripheral adrenergic inhibitors, and vasodilators. Cholesterol-lowering medications included statins, ezetimibe, bile acid sequestrants, PCSK9 inhibitors, and fibrates.

**Table 3:** Patient characteristics.

		Intervention Group Digital Lifestyle	Control Group Standard Care
		(*n* = 100)	(*n* = 70)
Demographics			
	Age at baseline, years	56.12 (7.32)	57.07 (9.94)
	Female, *n* (%)	49 (49.00)	32 (45.71)
Glycaemic control			
	HbA1c %	7.39 (1.20)	7.31 (1.27)
	HbA1c < 6.5%, *n* (%)	22 (22.00)	19 (27.14)
Lipids, mmol/ml			
	Total cholesterol	4.66 (1.28)	4.35 (1.13)
	LDL, median (IQR ^a^)	2.04 (1.06)	1.88 (0.97)
	HDL, median (IQR ^a^)	1.28 (0.45)	1.16 (0.38)
	TG, median (IQR ^a^)	3.21 (1.54)	3.04 (1.40)
Blood pressure, mmHg			
	Systolic	136.96 (15.24)	137.10 (16.06)
	Diastolic	88.26 (8.99)	86.91 (9.77)
Body composition			
	Weight, kg	104.24 (13.32)	103.68 (14.73)
	BMI, kg/m^2^	34.70 (3.29)	35.03 (4.40)
	Hip CF, cm	119.69 (9.70)	119.33 (10.92)
	Waist CF, cm	118.59 (9.91)	120.74 (9.61)
Medication, yes, *n* (%)			
	Glucose-lowering	80 (80.00)	57 (81.43)
	Lipid-lowering	62 (62.00)	35 (50.00)
	Blood pressure-lowering	54 (54.00)	33 (47.14)
Perception of life			
	Quality of life ^b^	0.81 (0.13)	0.77 (0.12)
	Mental well-being ^c^	25.04 (3.16)	24.64 (3.52)
Exercise, how often			
	Moderate ^d^	2.41 (1.22)	2.54 (1.34)
	Everyday ^e^	4.20 (1.76)	4.27 (1.67)
Diet, intake how often			
	Vegetables ^f^	2.68 (0.93)	2.71 (0.90)
	Fruit ^f^	2.17 (0.96)	2.68 (0.91)
	Fish ^f^	1.67 (0.86)	1.67 (0.86)
	Sweets ^f^	2.89 (1.09	2.59 (1.16)

Reported as mean (SD) unless otherwise noted, ^a^ interquartile range, ^b^ an index calculated based on the different dimensions measured in the European Quality of life—EQ-5D-5L. Index ranges from 0.35 to 1.0, ^c^ The Short-Warwick-Edinburgh Mental Well-being Scale—SWEMWBS. Index ranges from 7–35, ^d^ scored 1 (worst)—5 (best), ^e^ scored 1 (worst)—7 (best), and ^f^ scored 1 (worst)—4 (best) (Table S2).

**Table 4:** Between-group differences in changes from baseline to six months.

		N	Intervention Group Digital Lifestyle	N	Control Group Standard Care	Between Group Difference	*p*
			(*n* = 75)		(*n* = 38)	95% CI	
Weight							
	Mean change, kg (95% CI)	69	−4.24 (−5.49; −2.98)	37	−1.52 (−2.57; −0.48)	−2.71 (−4.56; −0.87)	0.005
	Mean change, % of baseline BW (95% CI)	69	−4.14 (−5.38; −2.90)	37	−1.47 (−2.44; −0.50)	−2.67 (−4.47; −0.88)	0.004
Proportion of BW loss							
	>3%, *n* (%)	69	36 (52.20)	37	8 (21.60)	30.60 (12.81; 48.30)	0.002
	>5%, *n* (%)	69	23 (33.30)	37	4 (10.80)	22.50 (7.56; 37.48)	0.011
	>10%, *n* (%)	69	8 (11.60)	37	0 (0.00)	11.60 (4.04; 19.15)	0.031
HbA1c							
	Mean change, % (95% CI)	75	−0.76 (−1.02; −0.49)	37	−0.61 (−0.85; −0.37)	−0.15 (−0.51; 0.22)	0.435
	Mean change, %, percent of baseline (95% CI)	75	−8.92 (−12.00; −5.84)	53	−7.27 (−9.87; −4.68)	−1.65 (−5.84; −2.54)	0.442
	Reduced from ≥6.5% to <6.5%, *n* (%)	62	24 (38.70)	40	8 (20.00)	18.70 (1.37; 36.05)	0.047
Body composition							
	BMI, kg/m^2^, mean change (95% CI)	69	−1.40 (−1.81; −0.98)	37	−0.51 (−0.85; −0.17)	−0.89 (−1.50; −0.28)	0.005
	Hip CF, cm, mean change (95% CI)	69	−5.64 (−7.10; −4.17)	37	−2.84 (−4.37; −1.31)	−2.80 (−5.05; −0.55)	0.016
	Waist CF, cm, mean change (95% CI)	69	−7.49 (−9.07; −5.91)	37	−4.06 (−5.84; −2.28)	−3.43 (−5.90; −0.96)	0.008
Lipids							
	Total cholesterol, mmol/mL, mean change (95% CI)	73	−0.32 (−0.56; −0.08)	49	0.06 (−0.24; 0.37)	−0.38 (−0.76; −0.01)	0.048
	LDL, median (IQR), mean change (95% CI)	56	0.23 (−0.01; 0.46)	39	0.40 (0.14; 0.67)	−0.17 (−0.53; 0.18)	0.330
	HDL, mmol/mL, mean change (95% CI)	74	−0.17 (−0.24; −0.10)	49	−0.05 (−0.13; 0.02)	−0.12 (−0.22; −0.02)	0.024
	TG, median (IQR), mean change (95% CI)	74	−1.06 (−1.39; −0.73)	49	−0.65 (−1.02; −0.28)	−0.41 (−0.91; 0.08)	0.105
Blood pressure							
	Systolic, mm Hg, mean change (95% CI)	69	−2.12 (−5.37; 1.14)	37	−3.49 (−7.89; 0.91)	1.37 (−3.99; 6.73)	0.617
	Diastolic, mm Hg, mean change (95% CI)	69	−2.26 (−3.92; −0.61)	37	−1.54 (−3.70; 0.62)	−0.72 (−3.42; 1.97)	0.601
Glucose-lowering medication use							
	Decreased or stopped, *n* (%)	74	11 (14.90)	41	1 (2.40)	12.40 ^a^ (3.05; 21.81)	0.015
	Increased or started, *n* (%)	74	2 (2.70)	41	7 (17.10)	14.4 ^a^ (2.27; 26.47)	0.021
Cholesterol-lowering medication use							
	Decreased or stopped, *n* (%)	74	1 (1.40)	41	2 (4.90)	3.50 ^a^ (−3.66; 10.71)	0.260
	Increased or started, *n* (%)	74	3 (4.10)	41	3 (7.30)	3.30 ^a^ (−6.08; 12.60)	0.460
Blood pressure-lowering medication use							
	Decreased or stopped, *n* (%)	74	0 (0.00)	41	1 (2.44)	2.44 ^a^ (−2.28; 7.16)	0.180
	Increased or started, *n* (%)	74	2 (2.70)	41	0 (0.00)	2.70 ^a^ (−1.30; 6.71)	0.290
Self-rated assessments							
	Moderate exercise, mean change (95% CI) Everyday exercise, mean change (95% CI) Eating Sweets, mean change (95% CI) Eating fish, mean change (95% CI) Eating fruit, mean change (95% CI) Eating vegetables, mean change (95% CI) Quality of life, mean change (95% CI)	75 75 75 75 75 75 75	0.62 (0.33; 0.90) 0.41 (−0.06; 0.88) 0.27 (0.05; 0.50) 0.37 (0.20; 0.54) 0.38 (0.15; 0.62) 0.49 (0.29; 0.69) 0.02 (−0.01; 0.05)	41 41 41 41 41 41 41	0.49 (0.10; 0.87) −0.08 (−0.62; 0.46) 0.46 (0.19; 0.73) 0.18 (−0.03; 0.39) −0.03 (−0.30; 0.25) 0.18 (−0.11; 0.47) 0.01 (−0.03; 0.04)	−0.12 (−0.61; 0.35) −0.48 (−1.25; 0.27) 0.18 (−0.18; 0.55) −0.19 (−0.47; 0.09) −0.41 (−0.79; −0.02) −0.31 (−0.66; 0.34) 0.01 (−0.04; −0.06)	0.600 0.210 0.310 0.180 0.040 0.080 0.553
	Mental well-being, mean change (95% CI)	75	−0.79 (−2.20; 0.62)	41	1.04 (−0.80; 2.88)	−1.83 (−4.06; −0.41)	0.115

^a^ Difference in percentage.

## Data Availability

The data presented in this study are available on request from thecorresponding author. The data are not publicly available due to restrictions on privacy.

## Supplementary Materials

The following supporting information can be downloaded at: https://www.mdpi.com/article/10.3390/nu14163424/s1, Table S1: Included variables, definitions, categories, and source, Table S2: Patient questionnaire on sociodemographic data and lifestyle habits at baseline and 6 months, and Table S3: Template of the Intervention Description and Replication (TIDieR) checklist (LIVA 2.0).
