# Supplementary material for: Reversing Type 2 Diabetes in a Primary Care-Anchored eHealth Lifestyle Coaching Programme in Denmark: A Randomised Controlled Trial
Source: Nutrients. 2022 Aug 19;14(16):3424. doi: 10.3390/nu14163424 (PMC9414066; doi:10.3390/nu14163424)
Supplement: Supplementary file 1 [file nutrients-14-03424-s001.zip › nutrients-1838931-supplementary.pdf]

**Table S1.** Included variables, definitions, categories, and source

| Characteristics | Definitions / measurements | Categories | Source |
|---|---|---|---|
| **Demographics and socioeconomics** | | | |
| Sex | Gender | (1) Male<br>(2) Female | Baseline face-to-face meeting with the health coach |
| Age | Age in mid-year | Continuous | Baseline face-to-face meeting with the health coach |
| Highest educational level attained | Highest educational level attained at the date of data extraction, based on the main groups in the Danish educational nomenclature with 13 educational groups based on years of education | Variable with three categories<br>(1) Primary education (<11 years)<br>(2) Middle high education (11-15 years)<br>(3) Higher education (16+ years) | Baseline face-to-face meeting with the health coach |
| Income level | Annual gross income in 2018 and employee benefits | Continuous variable or categorical variable with three categories<br>(1) DKK[a] 149,999 or less<br>(2) DKK 150,000-349,999<br>(3) DKK 350,000 or more | Baseline face-to-face meeting with the health coach |
| Civil status | Marital status | (1) Married or in civil partnership<br>(2) Unmarried<br>(3) Widow or longest living partner<br>(4) Divorced or cancelled partnership | Baseline face-to-face meeting with the health coach |
| Occupational status | Affiliation to the labor market | (1) Affiliated to the labor market (employed or self-employed)<br>(2) Unemployed (maternal leave or | Baseline face-to-face meeting with the health coach |

| | | | |
|---|---|---|---|
| | | job seeker allowance)<br>(3) Unemployed (unemployment benefit)<br>(4) Education<br>(5) Early retirement<br>(6) Retired<br>(7) Child | |
| Municipality | Municipality of residence | | Baseline face-to-face meeting with the health coach |
| **Clinical indicators** | | | |
| Blood pressure | Measured in seated position after 10 minutes of rest without speaking using an electronic Omron Automatic Blood pressure monitor device Model M3. Three measurements were performed one minute apart, and the lowest reported value was recorded | Continuous variable measured in mmHg | Baseline and follow-up meetings with the health coach |
| Blood samples (HbA1c[b], total cholesterol, LDL[c], HDL[d], TG[e]) | All levels were obtained using finger-stick sampling with a Hemocue HbA1c 501 Analyzer. The Hemocue analyser has the added advantage that HbA1c can be measured on non-fasting patients. A strict protocol was followed for the collection of blood samples. To ensure accuracy of the measurements, the Hemocue analyser was calibrated daily and in accordance with the manufacturer's user instructions. An additional calibration was carried out monthly using a special test kit to verify the sensitivity and accuracy of the measurements | Continuous variable measured in mmol/mol | Baseline and follow-up meetings with the health coach |
| BMI (Weight, height) | Weight (with clothes but no shoes and with 1 kilogram (kg) subtracted for clothing) divided by squared height (without shoes) both measured in accordance with the EHES guideline (http://www.ehes.info/manuals.htm) | Continuous variable measured in $kg/m^2$ | Baseline and follow-up meetings with the health coach |
| Waist and hip circumference | Measurement with tape measure around the waist (between lower rib and pelvic curvature) and hip | Continuous variable measured in cm | Baseline and follow-up |

| | | | |
|---|---|---|---|
| | (one hand above the inguinal medial line) in accordance with the EHES guideline (http://www.ehes.info/manuals.htm) | | meetings with the health coach |
| **Lifestyle habit indicators** | | | |
| Physical exercise | See appendix 2 - questionnaire | | |
| Diet | See appendix 2 – questionnaire | | |
| **Quality of life and mental well-being indicators** | | | |
| Quality of life (EQ-5D-5L) | Consists of five dimensions (1) Mobility (2) Self-care (3) Usual activities (4) Pain or discomfort (5) Anxiety or depression, with five possible levels of severity | Responses are coded as single-digit numbers expressing the level of severity selected in each dimension which are then coded into a score ranging from 0.35 to 1.0 | Questionnaire at baseline and follow-up meetings with the health coach |
| Mental well-being (SWEMWBS) | The SWEMWBS is a short version of WEMWBS, consisting of seven items covering subjective well-being and psychological functioning.[27] The seven items are (1) I've been feeling optimistic about the future (2) I've been feeling useful (3) I've been feeling relaxed (4) I've been dealing with problems well (5) I've been thinking clearly (6) I've been feeling close to other people (7) I've been able to make up my own mind about things | Each item is answered on a five-point Likert scale going from: none of the time, rarely, some of the time, often, all the time. The summary index ranges from 7-35. Higher scores indicate higher well-being and psychological functioning | |

[a] 1 DKK = 0.13 Euro (€) = 0,14 US$.
[b] HbA1c = hemoglobin A1c.
[c] LDL = low-density lipoprotein cholesterol
[d] HDL = high-density lipoprotein cholesterol
[e] TG = triglyceride

**Table S2**. Patient questionnaire on sociodemographic data and lifestyle habits at baseline and at 6 months

| **1. Select your highest completed level of education** | |
|---|---|
| None = No higher education | |
| Short = Short higher education of 2 years (ex. business academy) | |
| Medium = Medium-term higher education of 3-4.5 years (ex. BA level) | |
| Long = Long higher education of at least 5 years (ex. graduate or PhD level) | |

| **2. Select your marital status** | |
|---|---|
| Married or registered partnership | |
| Single | |
| Widowed or longest living partner | |
| Divorced or no longer in registered partnership | |

| **3. Select your employment status** | |
|---|---|
| On the labor market (employed or self-employed) | |
| Unemployed (maternity leave or on unemployment benefits) | |
| Unemployed (cash benefits) | |
| Studying | |
| Early retirement pension | |
| Retired | |

| **4. Have you been diagnosed with diabetes by your doctor?** | |
|---|---|
| Yes | |
| No | |

| **5. Are you taking medication for your diabetes?** | |
|---|---|
| Yes | |
| No | |

| **6. Do you smoke?** | |
|---|---|
| Yes | |
| No | |
| If yes, register number of cigarettes you smoke a day on average | |

| **7. How many items do you drink on a weekly basis? 1 beer, 1 glass of red wine / white wine, 1 glass of spirits are all each = one item. 1 bottle of red / white wine = 6 items. 1 bottle of spirits = 20 items** | |
|---|---|
| I do not drink alcohol | |
| Register number of items on a weekly basis on average | |

| **8. How often do you eat vegetables and / or root vegetables (fresh as well as frozen)?** | |
|---|---|
| Once a week or less frequently | |
| A few times during the week | |
| Once a day | |
| Twice a day or more often | |

| **9. How often do you eat fruit and / or berries (fresh, frozen, preserved, or as juice / smoothie)?** | |
|---|---|
| Once a week or less frequently | |
| A few times during the week | |
| Once a day | |
| Twice a day or more often | |

| **10. How often do you eat seafood as a main course?** | |
|---|---|
| A few times a month or less | |
| Once a week | |
| Twice a week | |
| Three times a week or more often | |

| **11. How often do you eat pastry, chocolate, sweets and / or drink soda?** | |
|---|---|
| Daily | |
| Almost every day | |
| A couple of times a week | |
| Once a week or less frequently | |

| **12. How much time do you spend per week on moderate physical exercise that makes you slightly short of breath? (ex. football, aerobics, tennis, jogging, biking for exercise)** | |
|---|---|
| 0 minutes | |
| Less than 30 minutes | |
| 30-59 minutes | |
| 60-119 minutes | |
| 120 minutes or more | |

| **13. How much time do you spend per week on physical activity? (ex. walks, gardening, cleaning, biking for transport)** | |
|---|---|
| 0 minutes | |
| Less than 30 minutes | |
| 30-59 minutes | |
| 60-89 minutes | |
| 90-149 minutes | |
| 150-299 minutes | |
| 300 minutes or more | |

**Table S3**. Template of the Intervention Description and Replication (TIDieR) checklist (LIVA 2.0)

| Item | Description |
|---|---|
| eHealth coaching sessions | Prior to the intervention, the health coaches receive training in setting SMART (specific, measurable, agreed upon, realistic, and time-based) goals with the participants using the eHealth solution LIVA 2.0 and in setting up action and coping plans that address barrier identification and problem solving. Participants in the intervention group have one or two personal meetings (face-to-face) with their health coach, followed by synchronous or asynchronous web-based consultations based on dialog by means of text or video. The consultations address the participant's registrations, goal setting, and questions regarding diet, exercise, and lifestyle plans, taking into consideration chronic diseases. The LIVA 2.0 app is set up with short explanations about different functions and notifications and reminders to the participants to register and give feedback about the health coaching. The sessions provide the participant with information in relation to their status, specific focus on goals, and recommendations on how to improve their behaviors.<br>Include BCT[a] from CALO-RE[b] taxonomy hereafter referred to as BCT) as follows: provide information on the consequences of the behavior in general and to the individual, goal setting, behavior and outcome, action planning, and barrier identification/problem solving; set graded tasks; prompt review of behavioral goals; prompt review of outcome goals; prompt rewards contingent on effort or progress toward behavior; prompt generalization of a target behavior; and provide feedback on performance. |
| Goals and inputs | Goals and inputs are always driven by the participant and are available to the participant, who can choose the focus area, set specific goals, and keep record of specified behaviors by reporting them on a daily, weekly, or monthly basis. This allows the user and the health coach to follow progress or setbacks as the numbers and registrations are visualized using graphs and curves. All coaching by the health coach follows national guidelines from the Danish National Board of Health. |
| Dietary goals and plans | Dietary goals and plans can be set at many different levels (eg, from simple changes aiming at changing one meal a day to more complex changes aiming at a completely new diet for the remedying of digestion problems). |
| Physical activity goals and plans | Physical activity goals and plans involve goal setting and recording of the type of physical activity and time for executing the given physical activity. The participant receives advice and/or a video on activities in a variety of contexts to foster physical activity as a more integrated part of life (BCT: provide instruction on how to perform the behavior, prompt generalization of a target behavior, and provide relapse prevention/coping planning). |
| Life goals | Goals on a healthy joyful life as the participant sees it (e.g., daily life with less stress, stronger social bonds with friends and family, coping skills for diseases, etc.). |
| Weight | Set the current weight and goal for a lower or higher weight and register new measurements on a daily, weekly, or monthly basis. |
| Steps | When downloading the LIVA 2.0 app, the participant can accept direct import of their information on steps recorded on a smartphone, and tailored messages on progress toward a set goal appear simultaneously (BCT: teach-to-use prompts/cues). Step count monitoring is encouraged but not required to enter the LIVA study. Some participants will have other ways of registering their physical activity level. |
| Pain, sleep, and mood | Give daily feedback on pain, sleep, and mood, which can affect the ability to perform a given behavior (BCT: relapse prevention/coping planning). |
| Smoking | Set goals to bring down the number of cigarettes smoked daily, leading to cessation. |
| Blood glucose and blood pressure | Keep a record of specified measures expected to be influenced by the different behavior changes addressed. In LIVA, this includes blood glucose and blood pressure measurements (BCT: prompt self-monitoring of behavioral outcome and provide information on consequences of the behavior in general and for the individual). |

| | |
|---|---|
| Forum | Online forum where the participant can exchange knowledge, gain social support, and build new relationships, and the health coach can provide advice to the participant (BCT: plan social support/change). |
| Coaching providers | Health coaches with basic training as nurses, physiotherapists, dieticians, or occupational therapists. In Denmark, all four education types consist of 420 European Credit Transfer System (ECTS) Points (3.5 years of full-time education). In addition to their education as health care professionals, they all undergo special training in using digital health coaching and practice digital health coaching for at least 2 years. |
| Coaching approach | Individually delivered via the app or web-based delivery. |
| Coaching location | Initial personal meetings in municipality health centers, general practice medical centers, or the research unit for general practice at the University of Southern Denmark or over the internet, and then, solely web-based delivery. |
| Coaching time and quantity | The initial consultation with a health coach is estimated to last for approximately 45-60 minutes. The subsequent synchronous or asynchronous eHealth coaching sessions are carried out once a week in the first 3 months and biweekly the next 3 months. |
| Tailoring | Every participant receives personalized eHealth coaching sessions from their designated health coach. The provided feedback is based on the participant's inputs on LIVA 2.0. |

[a]BCT: behavior change technique.

[b]CALO-RE: Coventry, Aberdeen, and LOndon-REfined taxonomy.
